# A missense mutation in *TRAPPC6A* leads to build-up of the protein, in patients with a neurodevelopmental syndrome and dysmorphic features

**DOI:** 10.1038/s41598-018-20658-w

**Published:** 2018-02-01

**Authors:** Hussein Sheikh Mohamoud, Saleem Ahmed, Musharraf Jelani, Nuha Alrayes, Kay Childs, Nirmal Vadgama, Mona Mohammad Almramhi, Jumana Yousuf Al-Aama, Steve Goodbourn, Jamal Nasir

**Affiliations:** 1 St. George’s University Hospitals, London, UK; 2 Princess Al-Jawhara Albrahim Centre of Excellence in Research of Hereditary Disorders, King Abdulaziz University, Jeddah, Saudi Arabia; 3 Department of Genetic Medicine, King Abdulaziz University Hospital, Jeddah, Saudi Arabia; 4 Institute of Infection & Immunity, St. George’s University of London, London, UK; 5 Institute of Neurology, University College London, London, UK; 6 Genetics Unit, Cell Biology and Genetics Research Centre, Molecular & Clinical Sciences Research Institute, St. George’s University of London, London, UK; 7 Present Address: School of Medicine, UCLAN, Preston, UK

## Abstract

Childhood onset clinical syndromes involving intellectual disability and dysmorphic features, such as polydactyly, suggest common developmental pathways link seemingly unrelated phenotypes. We identified a consanguineous family of Saudi origin with varying complex features including intellectual disability, speech delay, facial dysmorphism and polydactyly. Combining, microarray based comparative genomic hybridisation (CGH) to identify regions of homozygosity, with exome sequencing, led to the identification of homozygous mutations in five candidate genes (*RSPH6A*, *ANKK1*, *AMOTL1*, *ALKBH8*, *TRAPPC6A*), all of which appear to be pathogenic as predicted by Proven, SIFT and PolyPhen2 and segregate perfectly with the disease phenotype. We therefore looked for differences in expression levels of each protein in HEK293 cells, expressing either the wild-type or mutant full-length cDNA construct. Unexpectedly, wild-type TRAPPC6A appeared to be unstable, but addition of the proteasome inhibitor MG132 stabilised its expression. Mutations have previously been reported in several members of the TRAPP complex of proteins, including TRAPPC2, TRAPPC9 and TRAPPC11, resulting in disorders involving skeletal abnormalities, intellectual disability, speech impairment and developmental delay. TRAPPC6A joins a growing list of proteins belonging to the TRAPP complex, implicated in clinical syndromes with neurodevelopmental abnormalities.

## Introduction

Consanguinity, practised in many parts of the world, especially the Middle East, Turkey, Iran and North Africa, can reach rates of 40% or more, accounting for approximately half of all marriages. This phenomenon has been widely exploited by geneticists, using autozygosity mapping to identify disease related genes for both dominant and recessive conditions, in various isolated populations including the Amish^1,2^. However, prior to the discovery of single nucleotide polymorphisms (SNPs), the inherently high degree of homozygosity, itself presented a problem, with limited availability of markers to narrow down the regions of homozygosity, resulting in a lot of time and effort needed to screen the large numbers of candidate genes.

Recent developments in next-generation sequencing have led to an explosion in genetics research, leading to the discovery of large numbers of genes for obscure conditions, such as hairy elbows^3^, but require less time and resources compared to earlier approaches. However, the excessive level of genomic variation at the DNA sequence level, including *de novo* variation, can lead to many novel homozygous variants that cannot easily be narrowed down based on gene function alone.

We previously employed CGH arrays to identify regions of homozygosity and combined this with whole-exome sequencing (WES) to identify genes associated with complex neurological disorders^4–6^. Here, we report the identification of a large extended pedigree with intellectual disability (ID), facial dysmorphism, speech delay and developmental abnormalities, including postaxial polydactyly. We initially identified several regions of homozygosity. Subsequent analysis of WES data, corresponding to the homozygous regions, led to the identification of novel mutations in five candidate genes. These mutations appeared to be pathogenic and segregated in the family in an autosomal recessive manner, as expected. However, the mutations could not be prioritised, based on gene function alone. Working on the assumption that the disease related mutations lead to an unstable protein, we screened for the expression of mutant versus wild-type proteins expressed in HEK293 cells. We show this is a viable approach for functional screening of candidate genes for genetic disorders. We also discuss the potential role of modifier genes.

## Results

**Figure 1:**
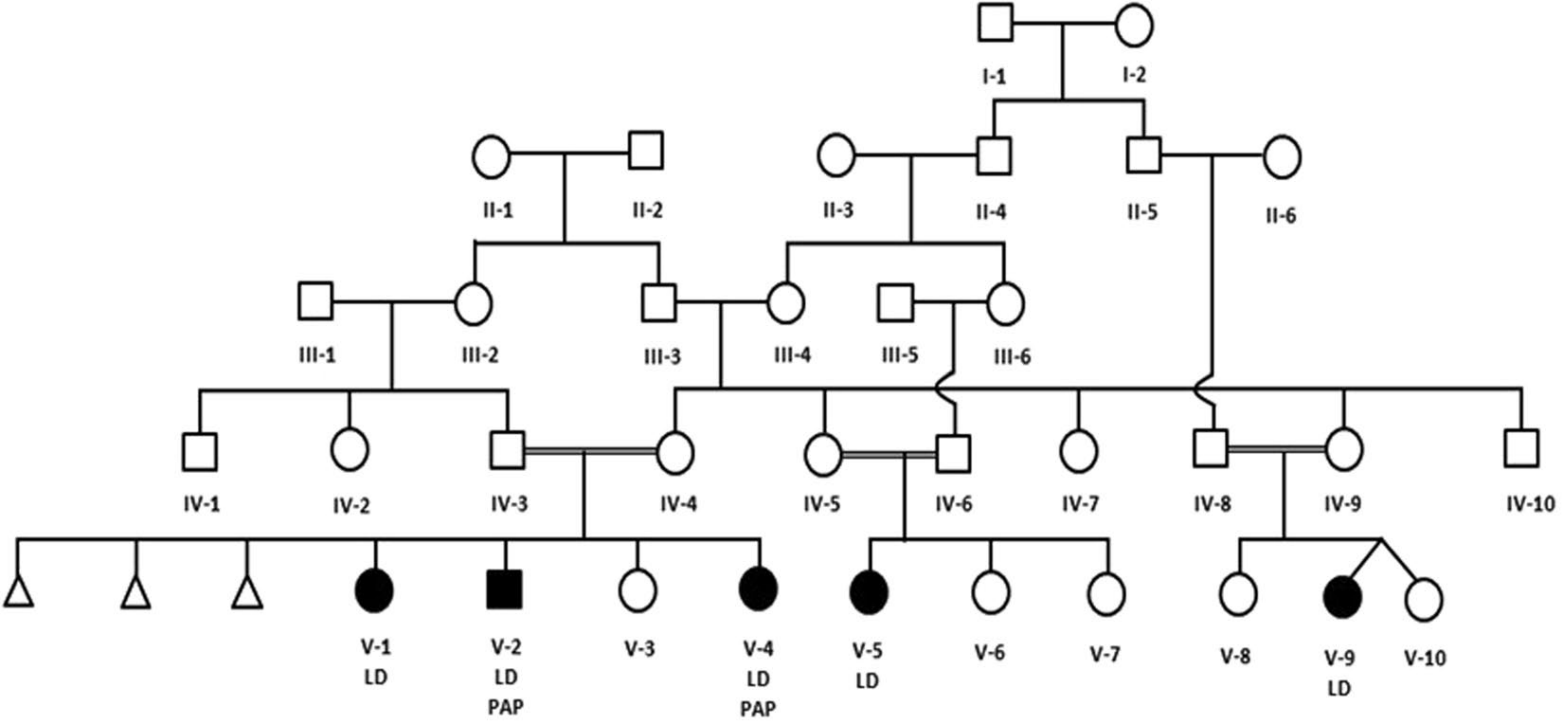
Pedigree diagram illustrating consanguinity. DNA samples from one branch of the family (from parents IV-3 and IV-4) with three affected children (V-1, V-2 and V-4) and one healthy child (V-3) were analysed. LD = Learning Disability. PAP = Postaxial Polydactyly.

We identified an extended pedigree from Saudi Arabia with multiple consanguineous relationships (Fig. 1). We identified three children with learning disability, speech delay, facial dysmorphism, postaxial polydactyly of the hands and bilateral 5^th^ toe clinodactyly, presenting as an autosomal recessive condition (Table 1). There were no concerns regarding their vision or hearing, and no complications were reported during pregnancy, labour or birth. The parents were first-degree cousins. They had one other healthy child and a history of three previous miscarriages (Fig. 1).

**Table 1:** Clinical summary. The clinical data for the three affected children is summarised. H = height in cm; W = weight in kg; HC = head circumference in cm.

Patient	Age	IQ	Skeletal abnormalities	PAP	Bilateral scar on ulnar side of both hands	Bilateral 5^th^ toe clinodactyly	Motor milestones achieved	Speech delay and LD	Growth	Dysmorphic features
V-1	12	68	Overlapping 2^nd^ toe over 1st			✓	✓	✓	H = 142 W 36.7 HC 57	Broad forehead Anteverted nares
V-2	11	67	Type A Postaxial polydactyly -both hands and feet	✓	✓	✓	✓	✓	H 131.5 W 28.3 HC 55	Broad forehead; Thick upper lip; large protruding ears;
V-4	7	73	Type A Postaxial polydactyly - both hands.	✓	✓	✓	✓	✓	H 112 W 20.4 HC 53.5	Large head; Broad forehead; 3 Café au lait spots

**Figure 2:**
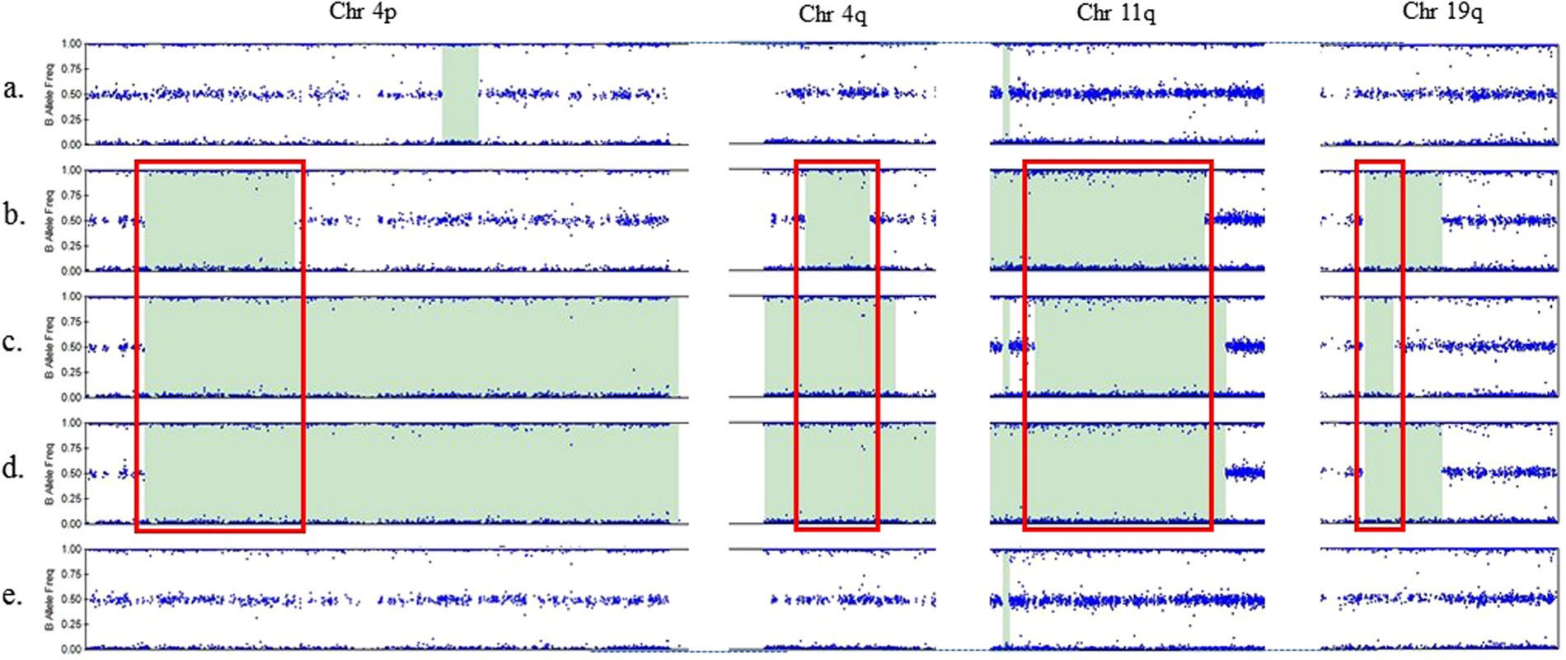
B allele frequency (BAF) (blue dots) is defined as the estimated number of B alleles divided by the sum of both alleles at a given SNP location. A BAF of 0 represents the genotype (A/A or A/−), 0.5 represents (A/B) and 1 represents (B/B or B/−). The green areas represent regions of homozygosity. The figure illustrates chromosomes 4, 11 and 19 with shared homozygous regions highlighted with a red box. Sample IDs: (**a**) mother (IV-4), (**b**) affected child (V-1), (**c**) affected child (V-2), (**d**) affected child (V-4), (**e**) father (IV-3).

We initially performed array CGH on five family members from one branch of the family (Fig. 1), including both parents (IV-3 and IV-4) and all three affected children (V-1, V-2, and V-4). This revealed four regions of homozygosity on chromosomes 4p15.31-4p15.2 (location: 22530016-30161847), 4q11-4q12 (location: 54762082-58098494), 11q14.3-11q23.3 (location: 90042539-117909101) and 19q13.31 (location: 45329214-47465366), shared by all three affected children but not the clinically unaffected members of the family (Fig. 2). Two of the affected children (V-2 and V-4) shared an extended region of homology on chromosome 4p, not present in V-1 (Fig. 2).

We next performed WES on one proband (V-1), using a commercial supplier (*Macrogen*). The data were filtered and sorted as previously reported^4–6^. We screened for novel stop-gain mutations, homozygous nonsynonymous SNPs and indels occurring at a frequency of less than 0.001, within the common regions of homozygosity identified by the array CGH array. Single nonsynonymous homozygous mutations in five potential candidate genes (*ALKBH8*, *AMOTL1*, *ANKK1*, *TRAPPC6A* and *RSPH6A*) were identified in the proband (Table 2). All the mutations were predicted to be pathogenic based on SIFT, PolyPhen2, MutationTaster and Provean analyses.

**Table 2:** Candidate genes resulting from the WES analysis. The chromosomal location, position and accession number of each gene is provided. The position of the mutation in the cDNA and protein sequence is also given.

Chr	Gene	Exon	Physical position	Chromosomal position	RefSeq accession number	c.DNA position	Amino acid change
11	*ALKBH8*	12	107375868	11q22.3	NM_138775	c.G1511C	p.W504S
11	*AMOTL1*	9	94592858	11q21	NM_130847	c.G2113A	p.A705T
11	*ANKK1*	2	113264421	11q22.3	NM_178510	c.A404C	p.H135P
19	*TRAPPC6A*	4	45667499	19q13.32	NM_024108	c.T319A	p.Y107N
19	*RSPH6A*	6	46299177	19q13.32	NM_030785	c.G2104A	p.E702K

**Figure 3:**
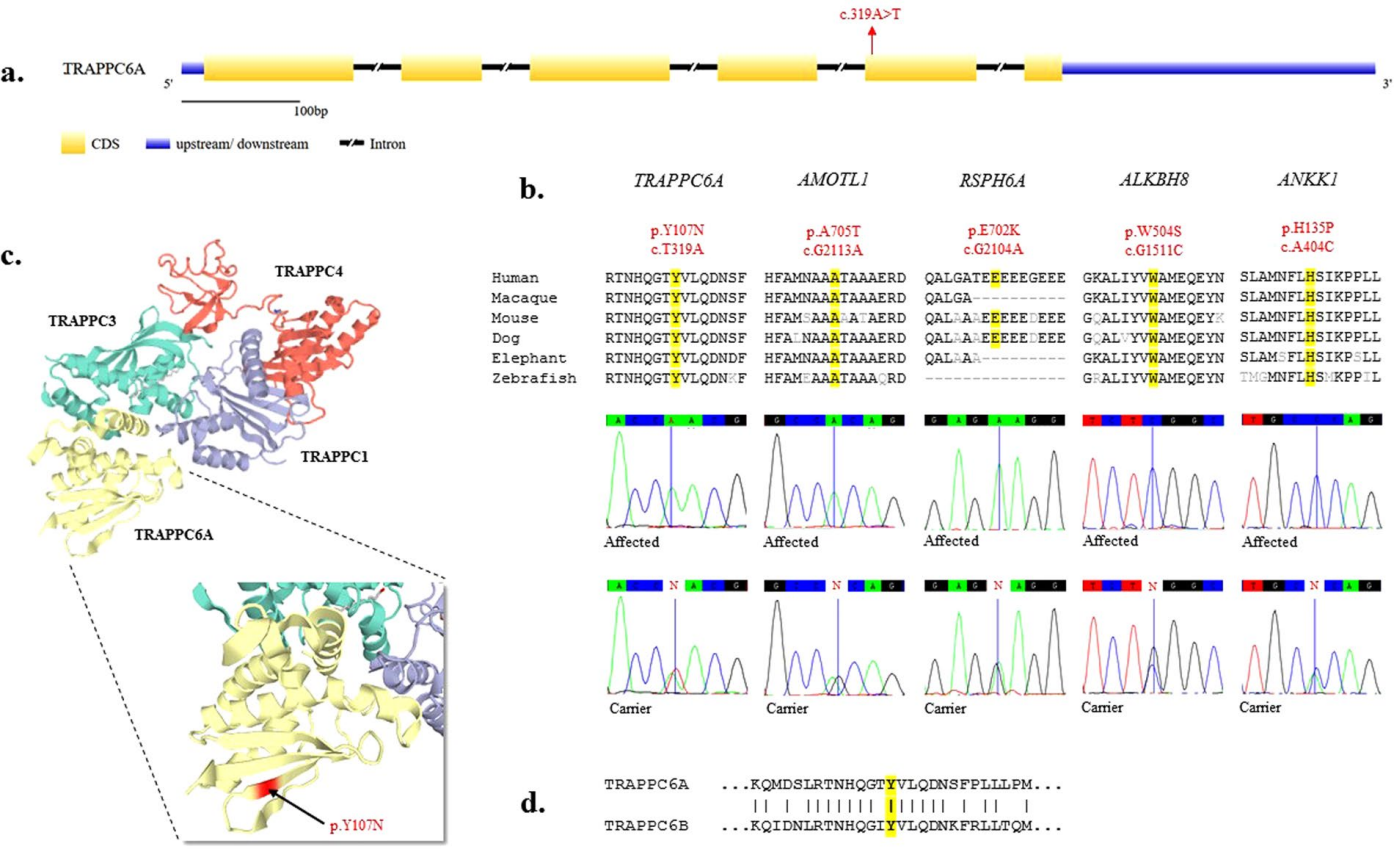
Gene structure, Sanger sequencing and protein modelling. (**a**) The structure of the *TRAPPC6A* gene is depicted showing intron-exon boundaries and the position of the mutation cT319A reported in this study. (**b**) Sanger sequencing of the five candidate genes (*TRAPPC6A*, *AMOTL1*, *ANKK1*, *RSPH6A*, *ALKBH8*) confirms the mutations. Alignment of the primary sequence across various species indicates the relevant residues are highly conserved. (**c**) The crystal structure of the TRAPP3-TRAPP6A-TRAPP1-TRAPP4 subcomplex is shown. TRAPPC6A forms a heterodimer with TRAPPC3 and interacts with other subunits to form TRAPP I, TRAPP II and TRAPP III complexes. The amino acid change is shown in red. Protein structure was generated using SWISS-MODEL^33^. (**d**) Alignment of TRAPPC6A and TRAPPC6B sequences shows the tyrosine residue in TRAPPC6A (p.Y107N) is conserved.

Sanger sequencing was performed on the affected, unaffected and parental DNA samples to confirm the mutations and to check for their correct segregation in this branch of the family (Fig. 3). All mutations showed correct patterns of segregation, corresponding to an autosomal recessive mode of inheritance, as expected. Sanger sequencing of DNA from 20 Saudi controls (40 chromosomes) and data from 40 in-house exome sequences, revealed they were homozygous for the wild-type allele for all five variants, ruling out a common population specific polymorphism. In addition, we screened various disease specific and population specific genomic databases, including dbSNP (http://www.ncbi.nlm.nih.gov/SNP/), 1000 genomes (http://www.1000genomes.org), Exome variant Server (http://evs.gs.washington.edu/EVS/), and Exome Aggregation Consortium ExAC (http://exac.broadinstitute.org/) data. ExAC alone represents data from over 60,000 genomes from different ancestries including, African, European, Latino and Asian. We also screened the recently described^7^ Greater Middle East (GME) Variome database (http://igm.ucsd.edu/gme/index.php), representing 2497 exomes.

All of the mutations we identified are either very rare, with allele frequencies of less than 0.002, or in the case of *ALKBH8*, absent in the named databases. Importantly, none of the mutations were found in the homozygous state, except for the *TRAPPC6A* (c.T319A) mutation. We identified an individual from the same general geographical location (Turkish Peninsula), with the same homozygous mutation, in the GME database which represents a collection of clinical samples enriched in neurodevelopmental disabilities^7^. We do not have any clinical information on this individual, but this individual is not homozygous for any of the other mutations we have described, in *ALKBH8*, *AMOTL1*, *ANKK1* or *RSPH6A*. We presume this patient might share common ancestry with our patients. Migration, population admixture, and very high rates of consanguinity resulting in a preponderance of recessive neurological conditions, are a common feature of this population^7–9^. Indeed, we previously reported a shared mutation in the *DDHD2* gene in two geographically isolated families from this region, suspecting common ancestry^5^.

To investigate the possibility that the mutations could result in an unstable protein, we generated full-length wild-type and mutant cDNA constructs in the mammalian expression vector pEF.V5.plink2^10^, using a PCR based strategy to amplify either the whole gene or segments of the gene to be assembled later. Mutant versions of these five constructs with the point mutations seen in patients were generated using a two-step PCR strategy with the mutation incorporated in the 5′ end of one of the primers in the first step of PCR (see Methods).

**Figure 4:**
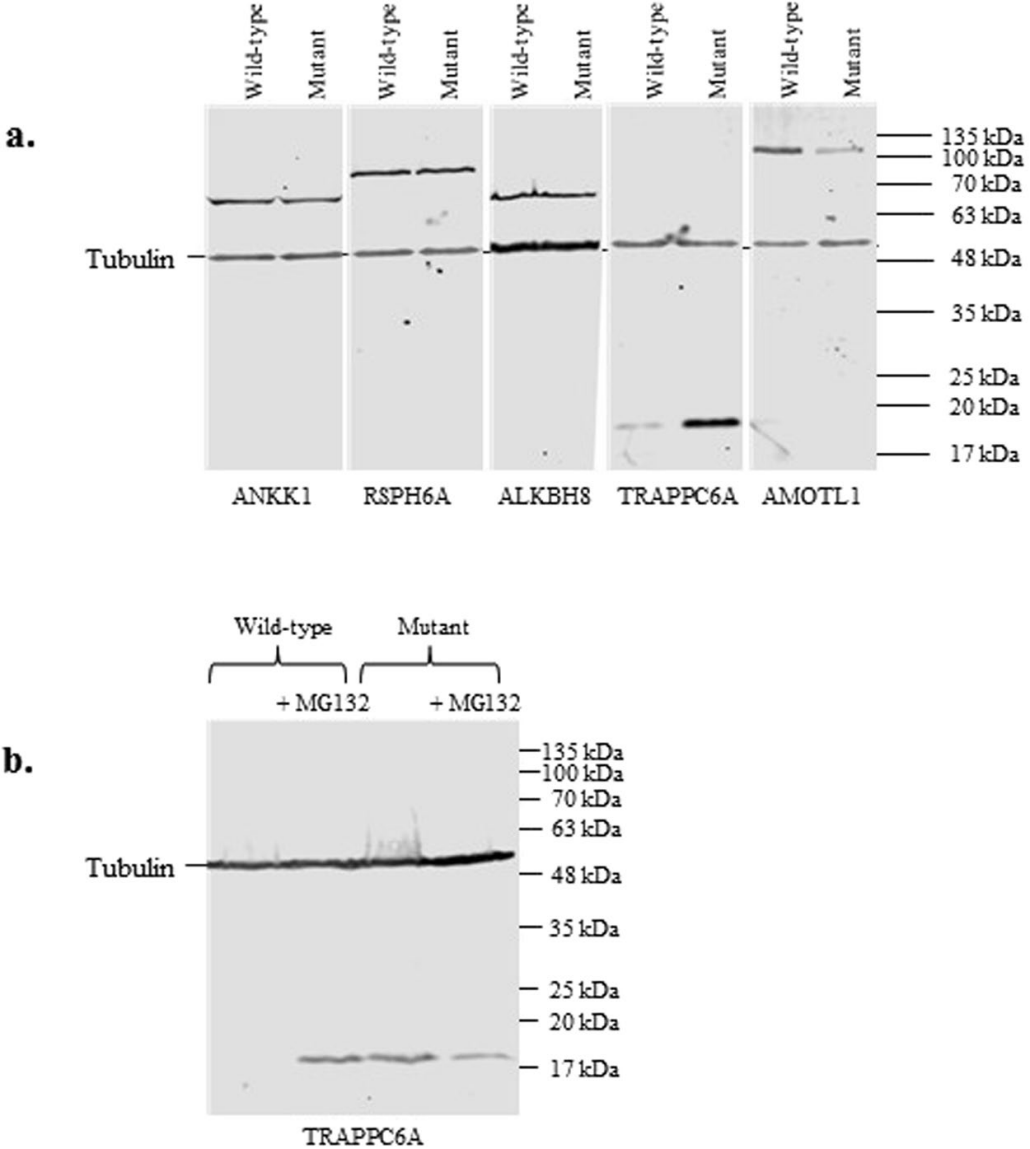
(**a**) Western blot of HEK293 cells transfected with constructs corresponding to the wild-type or mutant forms of all five proteins (AMOTL1, ALKBH8, RSPH6A, ANKK1, TRAPPC6A). (**b**) Western blots following addition of proteasomal inhibitor MG132 to HEK293 cells transfected with wild-type or mutant constructs for TRAPPC6A or AMOTL1. Tubulin is used as a loading control. A reference size ladder corresponding to the molecular weights is included. This figure was generated by cropping the original Western blots which are available on request.

The constructs were transfected into human HEK293 cells as described (Methods) and the proteins were detected by Western blotting using a mouse anti-V5 tag antibody (Fig. 4). A rabbit anti-alpha tubulin antibody was used to check for even loading across the lanes.

For three of these proteins (ALKBH8, ANKK1, and RSPH6A), both the mutant and wild-type forms were detected corresponding to the predicted molecular weight (Table 3), with no obvious differences in expression levels between wild-type and mutant proteins (Fig. 4a). However, the mutant form of AMOTL1 showed reduced expression compared to wild-type (Fig. 4a). Surprisingly, the mutant version of TRAPPC6A harbouring the pY107N mutation, appeared to be more highly expressed than wild-type, with negligible expression of the wild-type protein (Fig. 4a).

**Table 3:** Genes and Proteins Sizes. The length of cDNAs, the corresponding number of amino acids and the molecular weight of the protein for each gene is given.

Gene	V5-tagged ORF	Amino acids	kDa
*ALKBH8*	2040 bp	680 aa	70 kDa
*ANKK1*	2343 bp	781 aa	80 kDa
*RSPH6A*	2199 bp	733 aa	75 kDa
*TRAPPC6A*	567 bp	189 aa	20 kDa
*AMOTL1*	2916 bp	972 aa	100 kDa

The transfection was repeated for TRAPPC6A. Once again, only negligible amounts of the wild-type protein could be detected compared to the mutant, suggesting the normal protein might be inherently less stable or preferentially degraded. Given the widely-documented role of ubiquitination and proteasomal degradation in neurological conditions^11^, we repeated the experiment, but added the proteasome specific inhibitor MG132 to the cell culture media, 8 hours prior to harvesting. Addition of MG132 appeared to rescue TRAPPC6A degradation (Fig. 4b), indicating the wild-type protein is normally targeted for ubiquitination and proteasomal degradation.

## Discussion

Genetic studies of recessive disorders in consanguineous populations often yield multiple potential disease causing variants that all segregate with the phenotype and therefore cannot be easily excluded. In addition, the impact of a missense mutation on protein expression is often overlooked. Our study provides a viable strategy for screening multiple candidate genes by Western blotting, and suggests the general assumption that the mutant protein rather than the wild-type protein is unstable, should be treated with caution.

We identified five novel candidate genes for a childhood onset neurological condition with a distinct phenotype, characterised by intellectual disability, polydactyly, 5^th^ toe clinodactyly and speech and learning difficulties. The prospective mutations in each of the candidate genes are in highly conserved regions, segregating with the disorder in an autosomal recessive manner, and are all predicted to be damaging. However, it was not possible to prioritise them unequivocally, based on function alone. To further investigate the role of these genes in the disorder in this family, we decided to characterise them based on the level of protein expression of the wild-type versus the mutant forms of the proteins expressed in HEK293 cells.

*TRAPPC6A* (Trafficking Protein Particle Complex subunit 6A) encodes a subunit of the TRAPP complex which plays a role in ER-Golgi transport^12,13^. TRAPP, an approximately 670 kDa large protein complex, first discovered in yeast, consists of 11 subunits in mammals^14^, with a central core plus three sub-complexes (TRAPP-I, TRAPP-II and TRAPP-III). Interestingly, several members TRAPP complex family, have previously been implicated in genetic diseases^15^. For example, *TRAPPC2* is implicated in X-linked spondyloepiphyseal dysplasia tarda (SEDT), associated with skeletal abnormalities and short stature. *TRAPPC9* mutations result in intellectual disability associated with microcephaly and problems with speech^15^ and *TRAPC11* mutations cause a movement disorder, with ataxia, intellectual disability, and muscular dystrophy^16,17^.

Recently, homozygous splice mutations in *TRAPPC6B* have been reported in patients with a neurodevelopmental disorder with microcephaly, epilepsy and autistic features^8^ and a truncating loss of function mutation in this gene is associated with ID^18^. *TRAPPC6A* is closely related to *TRAPPC6B*, sharing 52% identity (data not shown). Both are part of the TRAPP-I sub-complex, but their corresponding genes map to separate chromosomes (chromosomes 19 and 14, respectively). The Y107 residue reported in this study corresponding to the *TRAPPC6A* mutation, is conserved in TRAPPC6B (Fig. 3d), indicating it is functionally important. In addition, the presence of the same cT319A homozygous mutation in *TRAPPC6A* in the GME database, which is enriched for genes involved in neurodevelopmental disorders, lends further support to the notion that the mutation in *TRAPPC6A* is the underlying cause for the phenotype we are reporting.

The pY107N mutation in *TRAPPC6A* results in substitution of a tyrosine for an asparagine. Tyrosine residues are a common target for phosphorylation and linked to signalling pathways in the CNS. Therefore, abrogating tyrosine phosphorylation, could potentially affect a range of downstream signalling pathways responsible for processes such as cellular growth, proliferation, survival and differentiation. In addition, phosphotyrosines are a key target for E3 ubiquitin ligase which facilitates proteasomal degradation of proteins^19^. We observed significantly reduced expression of wild-type TRAPPC6A compared to the mutant protein. However, we rescued the loss of expression of TRAPPC6A with the addition of the proteasome inhibitor, MG132. This supports the idea that TRAPPC6A is normally targeted for ubiquitination and rapidly degraded by the proteasome. We believe the mutant protein escapes this process, and builds up in the cell, disrupting the stoichiometry and assembly of the TRAPP complex. In addition, or alternatively, it might confer a toxic gain of function. This could be dosage dependent, which might explain why individuals heterozygous for this mutation do not have an associated phenotype. Interestingly, mutations in the *MAF* gene also lead to a build-up of the protein, resulting in impaired phosphorylation, ubiquitination and degradation of the mutated (unphosphorylated and unubiquitinated) protein and associated with a developmental syndrome with a distinctive facial appearance, sensorineural deafness, brachycephaly, intellectual disability, seizures and skeletal abnormalities^20^.

We observed reduced expression of the AMOTL1 (angiomotin-like protein 1) in HEK293 cells expressing the mutant version of the protein. Studies, including morpholino-mediated knockdown of *Amotl1* during zebrafish embryogenesis, and RNA–mediated silencing of *Amotl1* in mouse cells, show it can affect various processes such as remodelling of the actin cytoskeleton, cell polarity and endothelial cell migration^21^. AMOTL1 appears to be specifically involved in angiogenesis and cancer, potentially acting as a tumour suppressor or an oncogene. However, its role in a neurodevelopmental disorder, cannot be completely ruled out despite the fact homozygous loss of function mutations can be tolerated in humans without leading to a phenotype^22^ and heterozygotes individuals in our pedigree do not manifest with a phenotype. In addition, AMOTL1 could potentially interact with TRAPPC6A or act as a ‘modifier’ gene. Clearly, these aspects merit further investigations.

The expression levels of *RSPH6A*, *ALKBH8* and *ANKK1* were not affected by the mutation. RSPH6A (radial spoke head protein 6 homolog A), plays a role in ciliary function. Mutations in members of this gene family (*RSPH9* and *RSPH4A*) are specifically linked to recurrent respiratory infections and male subfertility due to impaired sperm motility^23^. ALKBH8 is thought to play a role in DNA repair based on its homology to a DNA repair gene found in *E. coli*. It is also involved in the modification of uridines, in the wobble position of certain tRNAs, which involves a methylation step^24^. This is believed to be important for increasing the efficiency of codon reading and preventing mistranslation. ALKBH8 is linked to sensitivity to DNA damaging agents and specifically bladder cancer, but interestingly, *ALKBH8*−/− mice do not display any obvious phenotype^24^. Ankyrin repeat and kinase domain-containing protein 1 (ANKK1), is a protein of unknown function that is specifically linked to neuropsychiatric disorders with impaired dopamine signalling, including schizophrenia, addiction, Tourette syndrome and ADHD^25^.

All three affected subjects have bilateral 5^th^ toe clinodactyly, but only V-2 and V-4 have postaxial polydactyly. V-2 and V-4 share an extensive region of homozygosity on chromosome 19 which is absent in V-1 (Fig. 2). This could contain a distal regulatory element or a modifier gene affecting *TRAPPC6A* expression leading to postaxial polydactyly. Similar features of modifier genes have been previously reported^26^. For example, the effects of varying genetic backgrounds on the *Ds* (disorganization) mutation in mice can lead to wide range of phenotypes, including polydactyly^27^.

A growing list of medical disorders involving intellectual disability (ID) is helping to shed new light on complex neurodevelopmental disorders, including neurological disorders, with polydactyly, pointing towards overlapping developmental pathways. For example, Bardet-Biedl syndrome (BBS) is characterised by postaxial polydactyly, cognitive deficits and speech delay. It is however genetically and clinically heterogeneous^28^ and affected by modifier genes^29^.

We shed new light on the role of another TRAPP complex protein (TRAPPC6A) potentially involved in a genetic disorder with neurodevelopmental features. Disorders of trafficking are common in neurological conditions^9,30^. In the case of the p.Y107N mutation, it is possible this disrupts the stoichiometry and assembly of the TRAPP complex. Further investigation on the effect of p.Y107N mutation on the structure, stability, subcellular localisation, expression and function of *TRAPPC6A*, and in particular its interaction with other TRAPP family members such as *TRAPPC6B*, could shed new light into cellular processes resulting in abnormal brain development and dysmorphic features and may also lead to new opportunities for therapeutic intervention.

In summary, TRAPPC6A potentially adds to a growing list of TRAPP complex proteins (TRAPPC2, TRAPPC9, TRAPPC11), including the closely related TRAPPC6B, involved in neurodevelopmental disorders. *TRAPPC6A* has been previously implicated in Alzheimer’s disease^31,32^. The p.Y107N mutation appears to affect the stability of the protein. We propose, the build-up of the mutant protein, could affects the stability of the TRAPP complex and negatively influence ER to golgi transport of proteins involved in developmental functions.

The homozygous mutation (c.T319A) in *TRAPPC6A* in a Saudi family reported in this study, is also present in one individual from the Turkish Peninsula, according to the GME database which is enriched for patients with neurodevelopmental disorders from the Middle East. Interestingly, this individual does not harbour any of the other mutations in *AMOTL1*, *ANKK1*, *ALKBH8* and *RSPH6A* identified in our study. However, our data need to be confirmed, as the contributions of AMOTL1, ANKK1, ALKBH8 and RSPH6A cannot be excluded on the basis of their protein expression and known biological functions alone.

## Methods

### Ethical approval

Prior to the commencement of this study, informed written consent was signed by the legal guardians, the parents, on behalf of the affected children, including agreement on publishing the research outcomes. The study was approved, according to the Declaration of Helsinki, by the Institutional Review Board (IRB) of the Princess Al-Jawhara Albrahim Center of Excellence in Research of Hereditary Disorders and the Unit of Biomedical Ethics Research Committee (ref. #24-14), Faculty of Medicine, King Abdulaziz University, Jeddah, Saudi Arabia.

### Clinical features

We present a consanguineous family with multiple affected individuals with learning difficulties, dysmorphic features and polydactyly.

The eldest (V-1) child was 12 years old at the time of examination. She achieved her motor milestones within the expected time. She presented with speech delay, learning difficulties and dysmorphic features. Her dysmorphological examination revealed, a large and broad forehead, anteverted nares, bilateral 5^th^ toe clinodactyly and overlapping of the 2^nd^ toe over the 1^st^. Her growth parameters revealed: height of 142 cm (just below 25^th^ centile), weight of 36.7 kg (below 25^th^ centile) and head circumference of 57 cm (above 97th centile). Her IQ test score was 68.

The second child (V-2) was 11 years old at the time of examination. He also presented with speech delay, learning difficulties and dysmorphic features. Soon after birth, postaxial polydactyly of both hands and feet were noticed, and surgical removal of bilateral hand polydactyly was performed during infancy. According to his mother, he achieved his milestones within the expected age range. He had a broad forehead, a thick upper lip, large protruding ears and bilateral 5^th^ toe clinodactyly. His growth parameters were: height of 131.5 cm (9^th^ centile), weight of 28.3 kg (on 2^nd^ centile) and head circumference of 55 cm (75^th^ centile). His IQ test score was 67.

The third affected child (V-4) was 7 years old at the time of examination. Soon after birth, she was found to have type A postaxial polydactyly of both hands, which were surgically removed at the age of 1 month. Like her siblings, she achieved her milestones within the expected age range. She too presented with speech delay and learning difficulties. She presented with a large head and broad forehead. Her growth parameters were: height of 112 cm (10^th^ centile), weight of 20.4 kg (below 10^th^ centile) and head circumference of 53.5 cm (almost on 98^th^ centile). Physical examination revealed bilateral 5^th^ toe clinodactyly, a scar on ulnar side of both hands and 3 café au-lait spots; 2 on her back and one on her abdomen. Her IQ test score was 73.

### Microarray and exome analyses

To investigate the possible involvement of chromosomal aberrations, and for the purpose of homozygosity mapping, we performed microarray analysis (*Illumina*, San Diego, CA, USA) on affected patients and their parents as previously described^5,6^. Whole exome paired-end sequencing, bioinformatic analyses and validation of the candidate genes by Sanger sequencing were also performed as previously described^5,6^.

### Preparation of plasmid constructs and mutagenesis

Plasmids containing full-length cDNAs for the genes of interest were obtained from *Source Bioscience* as follows: *AMOTL1* and *TRAPPC6A* were in the bacterial vector pOTB7 (GenBank accession numbers BC037539 and BC001907 respectively); *RSPH6A* was in pBluescript, (GenBank accession number BC057785); *ANKK1* was in the Gateway vector; pENTR223 (GenBank accession number BC156146) and *ALKBH8* was in the mammalian expression vector pCMVSport6 (GenBank accession number BC015183). The clone for *ALKBH8* encodes a short splice form of the *ALKBH8* protein, whereas the altered amino acid in the clinical sample resides in the long form; a full-length long form of *ALKBH8* cDNA was generated by commercially synthesizing the additional region (*Gene Art*; pMA-T:ALKBH8 exons) and religating into the shorter clone using compatible restriction sites. The open reading frames (ORFs) were amplified with primers containing the restriction enzyme sites for NcoI and EcoRI and inserted into the pEF.V5.plink2 vector, cut with these enzymes. The mutation was generated using a pair of back to back primers, with the desired mutation in the 5′ end of one of the primers. Two PCR products were generated from the wild-type construct using these primers. A second round of PCR (using the original primer pair) was performed to generate a single fragment flanked by NcoI and EcoRI sites, except for RSPH6A, flanked by SalI and XbaI restriction sites.

### Polymerase chain reaction (PCR)

PCR reactions were prepared using the Q5 High Fidelity DNA polymerase 2× Master Mix (*New England Biolabs*), 0.5 µM forward primer, 0.5 µM reverse primer, 20 ng of template plasmid and nuclease-free water up to 25 µl of total volume. The PCR program started with a 98 °C initial denaturation step for 10 minutes, followed by 98 °C for 15 seconds, 60 °C for 30 seconds, 72 °C for 1 minute for 20 cycles, followed by 72 °C for 10 minutes.

### Cell culture

Human embryonic kidney (HEK) 293 cells obtained from ATCC (cat# CRL-1573) were cultured in DMEM containing 10% foetal bovine serum (FBS) plus penicillin and streptomycin. 50 µl of each plasmid (diluted to 0.02 µg/µl in LBS - lactate buffered saline [20 mM sodium lactate, 150 mM NaCl, pH4.0]) was mixed with 50 µl 0.1 mg/ml linear polyethylenimine (PEI) MW~25,000 (*Polysciences Inc*, Warrington PA, USA) in LBS and incubated at room temperature for 20 minutes before adding 500 µl of Serum free media (SF-DMEM) and adding to the HEK293 cells in a 6-well plate. For cells expressing TRAPPC6A, MG132 (to a concentration of 25 μM) was added to the culture 8 hours prior to harvesting.

After 48 hours, cells were washed with PBS and lysed (on shaker for 10–15 minutes at room temperature) with 200 µl of cell lysis buffer (50 mMTris-HCl PH 7.4, 150 mM NaCl, 1 mM EDTA and 1% Triton X-100) containing protease inhibitors (1 mM Benzamidine, 30 µg/ml leupeptin, 5 µg/ml Aprotinin and 5 µg/ml Pepstatin A). The lysates were scraped, collected in Eppendorf tubes and centrifuged for 10 minutes at 4 °C. The supernatants were then transferred to new tubes. An equal volume of 2× SDS-PAGE loading buffer was added and the samples were heated to 95 °C for 2 minutes and immediately placed on ice.

### SDS-PAGE and Western blotting

The protein extracts were analysed by Sodium Dodecyl Sulphate-Polyacrylamide gel electrophoresis (SDS-PAGE) using a 10% resolving gel and a 5% stacking gel in 1% SDS running buffer (144 g glycine, 30.275 g Tris base, 10 g SDS, and fill up to 10 L with water) at 120 v for 1–2 hours.

Following electrophoresis, the gel was placed in cold transfer buffer (3.028 g Tris base, 14.41 g Glycine, 20% methanol) for 10 minutes. The separated proteins were blotted onto a low fluorescence hydrophobic polyvinylidene difluoride (PVDF) membrane (*immobilon*). The membrane was blocked with a 5 ml blocking buffer (TBS + 3% milk-no Tween) overnight at 4 °C on a shaker. After rinsing the membrane twice (with TBS + 0.1% Tween-20), primary antibody was added after dilution in 5 ml blocking buffer (TBS + 3% milk + 0.1% Tween-20) and incubated in room temperature for an hour on a rotator. It was washed three times (10 minutes each) with TBS + 0.1% Tween-20. The membrane was then incubated with the secondary antibody for an hour at room temperature in a dark setting (covered with foil paper) and washed twice (10 minutes each) in TBS + 0.1% Tween-20 and once in TBS without Tween-20, before scanning.

Blots were performed using a mouse anti-V5 tag antibody (a gift from Prof. Rick Randall, University of St Andrews), a rabbit anti-alpha tubulin antibody (*Abcam* ab176560) and either a goat anti-mouse secondary antibody (IRDye® 800CW, Li-Cor) or a goat anti-rabbit secondary antibody (IRDye® 680RD, Li-Cor) and analysed on a Li-Cor Odyssey CLx scanner.

### Data Availability

The datasets generated and analysed during the current study are available in the St George’s Research Data Repository (10.24376/rd.sgul.5787888.v1).
